# Physiological and modulatory role of thioredoxins in the cellular function

**DOI:** 10.1515/med-2022-0596

**Published:** 2022-12-12

**Authors:** Luis Omar Xinastle-Castillo, Abraham Landa

**Affiliations:** Departamento de Microbiología y Parasitología, Facultad de Medicina, Universidad Nacional Autónoma de México, Edificio A, 2o Piso. Ciudad Universitaria, Ciudad de México, 04510, México

**Keywords:** redox state, *S*-nitrosylation, signaling cascade, immune response, transcription factor

## Abstract

Thioredoxins (TRXs) are a class of ubiquitous and multifunctional protein. Mammal cells present three isoforms: a cytosolic and extracellular called thioredoxin 1 (TRX1), a mitochondrial (TRX2), and one specific in spermatozoids (TRX3). Besides, a truncated form called TRX80 exists, which results from the post-translational cleavage performed on TRX1. TRXs’ main function is to maintain the reduction–oxidation homeostasis of the cell, reducing the proteins through a thiol-disulfide exchange that depends on two cysteines located in the active site of the protein (Cys32-X-X-Cys35 in humans). In addition, TRX1 performs S-nitrosylation, a post-translational modification of proteins that depends on cysteines of its C-terminal region (Cys62, Cys69, and Cys73 in human TRX1). These modifications allow the TRXs to modulate the protein function and participate in regulating diverse cellular processes, such as oxidative stress, transcription, signaling cascades, apoptosis, inflammation, and immunologic response. This points out the crucial relevance of TRXs for cell function, signaling it as a strategic target for the treatment of many diseases and its possible use as a therapeutic factor.

## 1 Introduction

*Escherichia coli* thioredoxin (EcTRX) was initially identified as a hydrogen donor for the enzymatic synthesis of cytidine deoxyribonucleic diphosphate by the ribonucleotide reductase [1,2,3]. Currently, it is identified as the factor that restores the disulfide bonds of the active site of the peroxyredoxins 1 and 2 (PRX1 and PRX2) that eliminate the hydroperoxides (ROOH and H_2_O_2_) and peroxynitrites (ONOO^−^) produced in cell [1,4,5].

Thioredoxin (TRX) is a small protein with reduction–oxidation (redox) properties found in a wide variety of organisms from bacteria to humans. Mammal cells present several isoforms: a cytosolic thioredoxin 1 (TRX1) (≈12 kDa), a mitochondrial TRX2 (≈18 kDa), and one specific in spermatozoids TRX3 (≈13 kDa). Human TRX1 and TRX2 are the best described, both isoforms possess two cysteines in their active site (Cys32-Gly-Pro-Cys35), but only TRX1 possesses three more cysteines in its carboxylic region that participate in proteins nitrosylation (Cys62, Cys69, and Cys73). On the other hand, human TRX2 possesses an extra signaling sequence of 60 amino acids in the N-terminal that functions as a guide for its transport to mitochondria [1,2,4,6,7]. TRX1 is secreted despite not possessing a signaling peptide in its N-terminal, which indicates that it is not secreted through the endoplasmic reticulum and the Golgi apparatus (classical secretion route). This process is also mediated by a mechanism different from that observed for interleukin 1 beta (IL-1β), because TRX1 has not been founded in intracellular vesicles. Its specific mechanism has not been determined yet, but it has been demonstrated that the *in vivo* mechanism is sensitive to temperature and can be inhibited by unknown factors present in the serum [8,9,10,11].

This work shows the physiological processes where TRXs are involved and their modulatory role in cellular homeostasis and/or their involvement in the development of some diseases. Understanding the mechanisms of these processes points them out as possible candidates for the development of treatment strategies for diverse diseases.

## 2 TRX1 gene expression and protein structure

**Figure 1:**
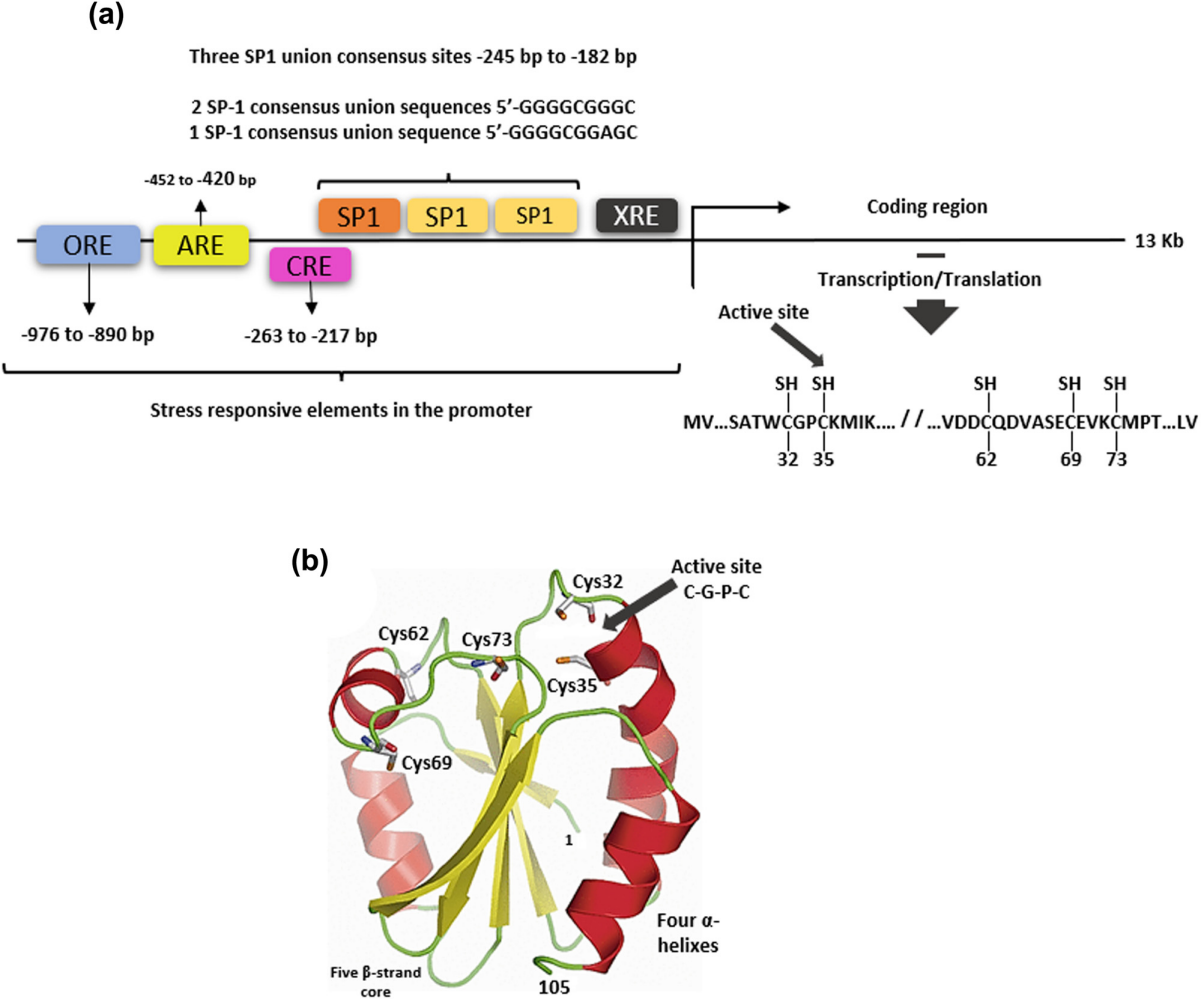
Human TRX1. (a) Gene structure showing the stress response elements in the promoter: ORE, ARE, CRE, XRE, SP1, and coding sequences where the important cysteines are located. (b) Model showing the typical structure of TRX1 with four helixes surrounded by five β-laminae, cysteines of the active redox site (Cys32 and Cys35) and cysteines that participate in transnitrosylation (Cys62, Cys69, and Cys73). PDB code: 1ERT.

The gene encoding human *TXN1* can be induced by a wide range of physicochemical stimuli, such as UV radiation, virus infections, the presence of hydrogen peroxide, and substances of metabolic origin, such as hemin, estrogen, prostaglandins, sulforaphane, and cyclic adenosine monophosphate (cAMP) [7]. It possesses a promoting region with diverse activation sites that respond to oxidative stress: an oxidative stress-responsive site element (ORE), an antioxidant-responsive element site (ARE), a cAMP-responsive element site (CRE), a xenobiotics-responsive element site (XRE), and three binding sites for the specificity protein 1 (SP1), responsible for gene activation in cellular processes, such as cell cycle regulation, apoptosis, and carcinogenesis. Of the latter, two binding sites share the same consensus sequence with a high binding affinity with SP1, whereas the third site possesses a slightly modified consensus sequence that also shows a high binding affinity with SP1 [7,9,12] (Figure 1a).

TRXs’ secondary structure is characterized by a β_1_α_1_β_2_α_2_β_3_β_4_β_5_α_4_ folding that is common for other proteins of the thiol-dependent antioxidant systems, such as the glutaredoxin, peroxyredoxin, and glutathione (GSH) peroxidase. This folding is divided into different domains: an N-terminal domain with β-folded laminae aligned in parallel; another C-terminal domain, with folded laminae aligned antiparallel; and a third element, a coil formed by the α_3_ helix that binds the N-terminal and C-terminal domains. Their tertiary structure is formed by five folded β-laminae constituting a nucleus surrounded by four α helixes [1,2,4,13] (Figure 1b).

## 3 TRX/TRX-Reductase system

**Figure 2:**
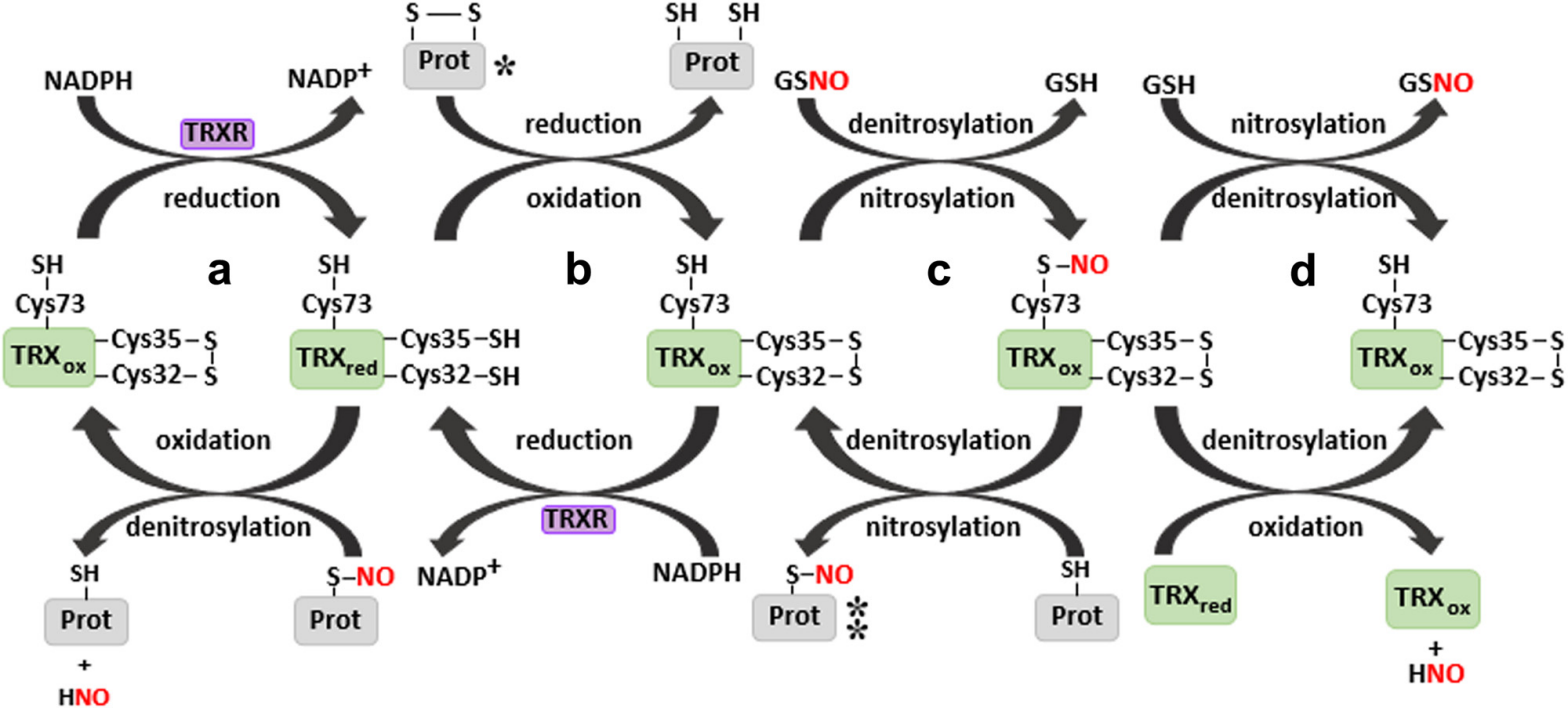
General mechanism of redox reactions and of transnitrosylation: TRX1 reduces its target proteins (b), TRX1 is reduced by TRXR at the expense of NADPH (a and b). TRX1 can reduce many targets (*), such as peroxyredoxins (PRX), the ASK1, the nuclear factor kappa-light-chain-enhancer of activated B cells (NF-κB), and interleukin 4 (IL-4). The oxidized TRX1 denitrosylates the nitrosylated glutathione (GSNO) releasing reduced GSH forming an oxidized-nitrosylated TRX (c). The latter returns to its denitrosylated form through the reaction with a second protein which is nitrosylated in the process (c). This mechanism performs as a regulator of the function of many proteins (⁑), such as caspases 3, 8, and 9, ASK1, the glyceraldehyde 3-phosphate-dehydrogenase (GAPDH), and the TRX1 itself. Finally, the oxidized-nitrosylated TRX can be denitrosylated by GSH or by a reduced Trx1 (d) through a mechanism like that observed in (a).

Among the main functions of TRXs are to act as a reducing factor of disulfide bonds; in this process, it undergoes an oxidation of the thiols present in the active site (Cys32 and Cys35 in the human TRX1), forming a disulfide bond between them and leading to the loss of its reducing activity. To recover its redox activity, it requires the thioredoxin reductase (TRXR) enzyme, which breaks the disulfide bond and reintegrates the thiol groups. TRXR is a homodimeric enzyme pertaining to the family of pyridine nucleotide-disulfide oxidoreductase. Its reaction mechanism with TRXs consists of a bimolecular nucleophilic substitution, which basically, transfers electrons from a reaction that starts with nicotinamide adenine dinucleotide phosphate (NADPH), passing over flavin adenine dinucleotide (FAD), to the active redox site of the TRXR, later, to the selenosulfide of the TRXR subunit, and, finally, to the disulfide of the oxidized TRXs [1,2,4,5,7,10,14] (Figure 2). There are two classes of TRXR; the first class corresponds to those of high molecular weight, with subunits of 55 kDa each and which are present in eukaryote cells. The second class corresponds to those of low molecular weight, with 35 kDa subunits, found in bacteria, plants, fungi, and unicellular eukaryotes. Structurally, both classes possess an active reducing site and binding domains to FAD and NADPH; however, those of low molecular weight lack the interphase domain that is present in the high molecular weight class. In mammal cells, three TRXR isoforms are present: one cytosolic TRXR1, a mitochondrial TRXR2, and one specific of testicles thioredoxin glutathione reductase (TGR). Structurally, TGR possesses the binding domains to FAD and NADPH found in both TRXR1 and TRXR2, but additionally, it possesses an extra glutaredoxin domain in its N-terminal. In general, the structure of TRXRs is like that of the GSH reductase, except that the TRXR possesses a four-amino-acid extension in the C-terminal (Gly-Cys-Sec-Gly) [2,4,14].

The relevance of TRXs for the correct cellular function is in examples like the *Trx1* gene knockout murine model, where its absence causes lethal effects during early stages of development and in the morphogenesis of embryos. In contrast, overexpression of *Trx1* confers a higher resistance to oxidative stress and an increase in life expectancy, pointing out the relevance it has in cell survival processes. Another example is the overexpression of *Trx1* in the murine model of cardiac diseases, where its relevant role in essential processes for cardiac function has been demonstrated, participating in glycolysis, tricarboxylic acid cycle, beta-oxidation, the function of the mitochondrial permeability transition pore (mPTP), and the contractile apparatus of myofibrils. In the latter, oxidation of contractile proteins is the origin of the loss of plasticity and contractility of the cardiac muscle. It must be mentioned that the administration of TRX1 in these cases reverses the oxidative process and allows the cardiac muscle to recover its function [6,7].

## 4 Regulation of the redox homeostasis

Aerobic cellular metabolism induces the constant exposure to reactive oxygen species (ROS) and reactive nitrogen species, for example, those produced by the passage of electrons through the different constituents of the mitochondrial respiratory chain and those produced by phagocytic cells of the immune system. The existence of reactive species inside the cell may imply a risk factor for the integrity and cell function because of their high reactivity degree. Nevertheless, the permanence of these reactive species at low concentrations works as a regulator of important processes, such as cellular proliferation and survival. This regulating effect has its origin in the chemical modifications caused in proteins, among them stand out those undergone by some cysteines, the least abundant of the amino acids, and whose chemical properties allow them to significantly influence the function, structure, and activity of proteins. These modifications are diverse and include, mainly, oxidation processes of the thiol group forming sulfenic acids (–C–SOH) capable of forming inter- and intramolecular disulfide bonds, sulfinic acids (–C–SO_2_H), sulfonic acids (–C–SO_3_H), S-nitrosylations (–S–NO), and glutathionylations (–S–SG) [1,7,10,15,16] (Figure 2).

High oxygen/nitrogen reactive species concentrations act as unspecific oxidants of many cell components, among them lipids, proteins, and nucleic acids. Because of the latter, it is very important for the cell to maintain a strict redox steady state of these reactive species, ensuring their correct function. To accomplish this task, there are enzymatic and protein mechanisms in charge of transforming the reactive species into less reactive and innocuous compounds for the cell. These detoxifying systems are essential for cells; hence, the diminution or inactivation of these systems results in the accumulation of reactive species that can lead to cell death [7,15,17]. The global process of ROS elimination in the cell is performed by different systems that can be categorized according to the reducing molecule needed, i.e., the TRX-dependent ones, the glutaredoxin-dependent ones, and the GSH-dependent ones. Of these, the TRX- and GSH-dependent ones represent the two main antioxidant systems. GSH and GSH peroxidase participate in functions that seem to overlap those of TRXs; nevertheless, it is proposed that both systems participate in parallel and constant communication. The link between both redox systems is formed due to the capacity of the GSH system to function as a backup pathway for the reduction of the oxidized TRXs, contributing to maintain the correct concentration of the reduced TRXs, a reduction process that is generally accomplished by the TRXR. On the other side, TRXs can function as proteins that restore the oxidation state of the GSH, establishing a backup mechanism between both pathways [4,15,17].

The reducing capacity of TRXs is the main reason that allows them to participate actively in the regulation of many cellular processes. Under conditions of high oxidative stress, susceptible proteins and enzymes are oxidized at specific sites inducing alterations in their functions and, in occasions, their complete inactivation. In these cases, TRXs function as reducing factors that allow proteins to recover their functional state at the expense of the oxidation of the TRX itself [1,4,13,16,17,18,19,20] (Figure 2). The adenine nucleotide transporter 1 (ANT1) provides an example of TRXs modifying cell processes due to their redox activity. The ANT1 plays a central role within the process of supplying energy to the cell by enabling the electrogenic exchange of ATP and ADP between the mitochondrial matrix and the intermembrane space. It also participates in the opening of a protein complex called mPTP, which allows the diffusion of molecules smaller than 1.5 kDa and which is a mediator of the necrotic and diffusion process by the intrinsic pathway [6,21,22,23,24]. Although the composition of the mPTP is not well defined, it has been shown that ANT is an important component for the mPTP function; complete deletion of ANT isoforms (ANT1–ANT4) results in desensitization of the mPTP [21,22,23,24,25]. In this sense, ANT1 has proven to be a protein susceptible to oxidation in one of its cysteines (Cys159 in humans and Cys160 in mice), suggesting that TRX1 functions as a protector of the redox state of ANT1 [6].

A couple of examples that illustrate the importance of maintaining the correct function of ANT1 are in Parkinson’s disease, characterized by the loss of dopaminergic neurons within the *substantia nigra pars compacta* (sNpc), which contributes to the deterioration of motor functions. The origin of this neurodegeneration is in the presence of abnormally folded protein aggregates, mainly α-synuclein. In this context, it has been hypothesized about the relationship between the formation of these aggregates and the decrease in the soluble ANT1 protein in the brain. Experimental results have also shown the presence of α-synuclein and ANT1 in the cerebral cortex of mice with the location of ANT1 in the center of these aggregations, suggesting that the phenomenon of aggregation of α-synuclein has ANT1 as an important factor [26]. The second example is the immunological function of ANT1 in regulating the IL-6 expression of macrophages; the mechanism involved is carried out at transcriptional level by decreasing the phosphorylation of JNK, one of the main signaling pathways involved in accelerating the transcription of inflammatory cytokines [24]. One more example is that described in the study of Parkinson’s disease, where the susceptibility of the protein deglycase DJ-1 to undergo changes in its redox state has been identified. DJ-1 is a multifunctional protein capable of regulating the transcription of genes, involved in signal transduction, in the elimination of ROS, and having chaperone functions in mechanisms against oxidative stress. The loss of its function causes neurons to become more sensitive to oxidative stress and increases neuronal apoptosis. Studies suggest that human TRX1 could function as a partial protector of the redox state of the DJ-1 protein because one of its four cysteines (Cys53) is a potential target for the reducing action of TRX1 [6].

The potential of TRXs as regulators of different cell processes is evidenced even better with assays studying the possible reduction sites in proteins. The development of this type of technique in plants has yielded a good number of potential targets for TRX1, with proteins as diverse as those involved in ion channels, stress-responding proteins, and metabolism components (glycolysis, carboxylic acids cycle, oxidative phosphorylation, and beta-oxidation). These results allow assuming an equally important effect in animal models and not only in plants [6]. In humans, the main TRX1-secreting cell groups are the monocytes, activated B-lymphocytes, lymphocytes, liver cells, and fibroblasts. Its expression is also carried out on the surface of some cells, such as endothelial and leukemia cells, in which TRX1 is found in its reduced form [11,27].

## 5 *S*-Nitrosylation of proteins

Through its redox activity, TRX1 can regulate different and very varied cell mechanisms. However, the presence of thiol groups, different from those located in the cysteines of the active site, grants it other regulating capacities through a reversible post-translational modification mechanism known as S-nitrosylation. Among the main cellular mechanisms regulated by S-nitrosylation are apoptosis; this results from the nitrosylation of cysteine residues in caspases 3, 8, and 9, the glyceraldehyde-3-phosphate dehydrogenase (GAPDH), the apoptosis signal-regulating kinase 1 (ASK1), the B-cell lymphoma 2, p53, FAS, and TRX1 itself [28] (Figure 2c).

The global nitrosylation process of proteins is dependent on several factors, such as the presence or permanence of nitric oxide (NO) in the cellular environment, the concentration of this specie, location of the stimulus, the cellular moment at which the stimulus occurs, and the equilibrium between the nitrosylation and denitrosylation processes. The latter explains why, sometimes, the effects derived from the NO presence can be opposite, functioning in some conditions as an apoptotic signal and in others as an inhibitor signal of apoptosis. As main sources of intracellular NO are three isoforms of the NO synthetase: a neuronal isoform, an endothelial one, and an inducible one. [7,28]. Because the nitrosylation process causes changes in the performance of proteins, the cell needs to count upon denitrosylation systems that will allow reversing those modifications. This process is mediated mainly by two mechanisms: one non-enzymatic where the nitrosyl (NO–) group is transferred to small molecules, for example, GSH and TRXs, and the second mechanism of enzymatic origin where systems like that of *S*-nitroglutathione reductase participate; the latter is a transnitrosylator of low molecular weight molecules. The transnitrosylation reaction carried out by TRX1 depends on the thiol groups present in both the TRX1 and the target protein, accompanied by the important fact that, in the process, TRX1 is also regulated in its function because of its nitrosylation [7,28] (Figure 2c).

In normal cell conditions, a low protein nitrosylation rate is maintained, although it can increase in response to diverse extracellular stimuli and, under certain conditions, proteins nitrosylation can occur constitutively, which alters the correct function of proteins. Examples of this phenomenon are chronic-degenerative diseases, such as cardiovascular diseases, cerebrovascular events, neurological alterations, such as Parkinson’s and Alzheimer’s diseases, and metabolic diseases, such as type 2 diabetes and obesity [29]. Studies on the nitrosylation processes dependent on the human TRX1 identified Cys79 as the main responsible for the transnitrosylation processes, being caspase 3 as one of the primary transnitrosylation targets. The capabilities of TRX1 vary in the function of its redox state: its oxidized and nitrosylated form functions as a transnitrosylase of caspase 3, which inactivates and inhibits apoptosis through this route. In contrast, TRX1 in its reduced state induces denitrosylation of caspase 3 allowing for its activation [28].

Another protein that is the target of nitrosylation by TRX1 is GAPDH; when the latter is nitrosylated, it becomes associated with a series of proteins that allow for its translocation to the nucleus; this complex stabilizes and activates a protease deriving in apoptosis. One more example is found in the nitrosylation of Cys869 of human ASK1, and this modification causes a diminution in the ASK1 affinity for its substrate (MAPK kinase 3 and 6), interfering in the signaling process of its corresponding pathway [28]. In addition, the nitrosylation of TRX1 becomes relevant within the apoptosis activation pathway, because it modifies its capacity to bind to ASK1, which causes dissociation of the ASK1/TRX1 complex, thereby allowing ASK1 to trigger the apoptosis signaling cascade [28].

## 6 Degradation of proteins modified by redox changes

Protein degradation regulated by TRX1 occurs through several mechanisms that are dependent on its reducing activity. The first mechanism involves alteration in the ubiquitination process, which is a post-translational modification of proteins that depends on the binding of small ubiquitin polypeptides to the protein; in this case, TRX1 induces rupture of the thioester bindings between the ubiquitin and the thiol groups of proteins E1 and E2, interfering with the degradation process of proteins. In the second mechanism, TRX1 modifies the signals associated with the degradation of proteins, such as oxidation of specific cysteines inside the structure of proteins, avoiding the degradation of proteins. The third mechanism involves the modification of the activity of the proteasome 20S subunit, because this subunit is susceptible to the changes in the redox state and its activity depends on the presence of two disulfide bonds susceptible to be reduced by the action of TRX1, causing inactivation of that subunit. Another mechanism involves the lipid-phosphate phosphatase, phosphatases, and deubiquitinating enzymes, which possess cysteine residues that are critical for their catalytic functions and are also susceptible to oxidative processes. In this case, the TRX1 restores the redox state of the cysteines, which allows the proteins to recover their functionality. The reintegration process of the redox state of methionines gives a last example, once they have been oxidized to methionine sulfoxides, a process accomplished by the methionine synthase reductase and which is a target for the reducing action of TRX1 [4,15,17].

## 7 Regulation of apoptosis

**Figure 3:**
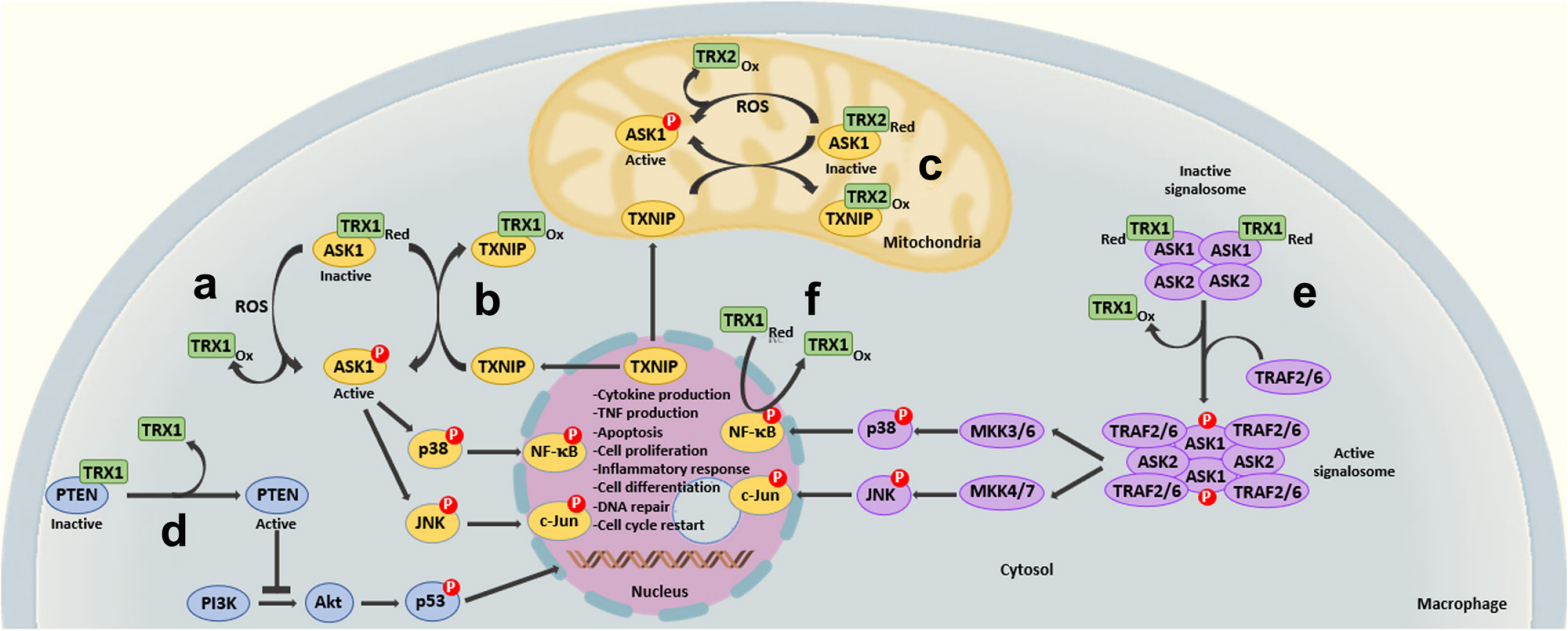
TRX1 as a regulator of different processes in macrophages. (a) Reduced TRX1, through any of its cysteines of its active site binds to a cysteine of the N-terminal end of the ASK1, hindering its autophosphorylation and activation. A high stress oxidizes TRX1 separating it from ASK1, which induces activation of the transcription factors nuclear factor kappa B (NF-κB) and c-Jun (involved in the inflammatory response and signaling of cytokines and growth factors). (b) The reduced TRX1 binds the TXNIP through the formation of a disulfide bond between Cys32 of TRX1 and Cys247 of TXNIP, releasing it and activating ASK1 (involved in apoptosis). (c) The regulation process of ASK1 is also performed in the mitochondrion but with the participation of an isoform of TRX (TRX2). (d) The reduced TRX1 binds the phosphatase and tensin homolog (PTEN) forming a disulfide bond between the Cys32 of TRX and the Cys212 of PTEN, inactivating PTEN. The reduction of this disulfide bond induces the release and activation of PTEN, which blocks the signaling cascade that activates the transcription factor p53 (apoptosis, cell proliferation, and angiogenesis). (e) The capacity of TRX1 to bind to ASK1 allows it to be a regulator of the ASK1 signalosome. The dissociation of TRX1 from the inactive signalosome causes ASK1 to autophosphorylate, activate, and recruit the proteins tumor necrosis factor type 2 receptor-associated protein 2 and 6 (TRAF2/6). This activates the ASK1 signalosome and the transcription factors NF-κB and c-Jun. (f) Finally, TRX1 exerts a direct transcription regulating mechanism by reducing the p50 subunit of NF-κB, inducing changes in the affinity of the protein for DNA.

Regulation of apoptosis by TRX1 is a well-described mechanism and shown to be extremely relevant for cell survival. As part of this mechanism, it is fundamental to know the function of the main regulator of the TRX1 activity, a protein known as thioredoxin-interacting protein (TXNIP), also called TRX-binding protein 2 or vitamin D3 up-regulated protein 1. TXNIP, through the reaction of its cysteines (Cys24 and Cys63 in human TXNIP), binds to the cysteines of the active site of TRX1, forming disulfide bonds between both proteins; this inactivates TRX1 and hinders it from becoming a substrate for TRXR. Under normal conditions, and in the absence of TXNIP, the reduced form of TRX1 binds to ASK1 protein, maintaining the latter inhibited and interrupting the signaling route for apoptosis. In turn, when oxidative stress prevails, TXNIP (usually confined to the nucleus) mobilizes to the cytosol and mitochondrion, its presence causes the binding to TRX1, which releases ASK1 that can now be activated to trigger the signaling route for cell apoptosis [4,14,30] (Figure 3). Besides the regulating role exerted by TXNIP during apoptosis, it is also related to energy metabolism. In experimental models, lack of TXNIP predisposes to death due to severe bleeding and induces important metabolic alterations, such as hypoglycemia and liver steatosis during intermittent fasting. This could be indicating more cellular mechanisms, where the TRX1 activity could be key to understand these phenomena [7].

## 8 Regulation of transcription

Another important regulation mechanism carried out by TRX1 takes place during the genetic transcription process, where it causes changes in the redox state of some transcription factors, modifying their affinity for the DNA and affecting the expression of its target genes. Among the involved factors are the nuclear factor kappa-light-chain-enhancer activated B cells (NF-kB), the activator protein 1 (AP-1), p53, the polyomavirus enhancer-binding protein-2 (PEPB-2), the redox-factor 1 (Ref-1), the hypoxia-inducible factor 1-alpha (HIF-1), the histone deacetylase 4, and the glucocorticoids receptor. Of these, NF-kB and AP-1 are of interest because they influence the production of inflammatory molecules in macrophages [6,7,14,18,20,28,31,32]. The NF-κB is a heterodimer formed by two proteins, of which the most important subunits are p50 and p65. Both contribute to the DNA-binding specificity, but subunit p50 is susceptible to changing its DNA affinity because of changes in the redox state of one cysteine located in the binding region of DNA (Cys62 in human), and its reduction derives in an increase of DNA affinity [20,31] (Figure 3).

Besides, to the direct regulation mechanism of the NF-κB activity by TRX1, there is a second mechanism that causes the activation of some components within the MEKK1/JNK signaling cascade is the degrading IκB that is an inhibitor of NF-κB. The degradation allows the release and translocation of NF-κB to the nucleus, activating the transcription of genes through its interaction with factors, such as Ref-1, HIF-1, p53, AP-1, glucocorticoid receptors, and estrogen receptors. Regarding the cell response to estrogens, TRX1 acts like a factor that helps the estrogen receptor α (Erα) in its regulating role of genes expression; this regulation depends on the direct interaction between TRX1 and Erα [7,14,15]. Another example of transcriptional regulation is with the histone diacetylase 4, which is a target of TRX1 in the experimental model of murine cardiac disease. Here, TRX1 fosters the formation of a protein complex in charge of the nuclear translocation of the histone deacetylase 4, regulating indirectly the expression of anti-apoptotic genes [6].

## 9 Activation of signaling cascades

TRX1 also acts on proteins that participate in the mitogen-activated protein kinase (MAPK)-dependent signaling cascades, which are highly conserved in all eukaryote cells. MAPKs are proteins in charge of performing specific phosphorylations in serines and threonines of their corresponding target proteins; the classic signaling route includes the participation of three proteins that perform phosphorylation in sequence; in the first step, a MAPK kinase kinase (MAP3K) phosphorylates and activates a MAPK kinase (MAP2K), then this MAP2K phosphorylates and activates a MAPK. Among the signaling pathways dependent on this mechanism are those that converge in the extracellular signal-regulated kinase (ERK), the c-Jun N-terminal kinase (JNK), and the p38 [7,14,15,24,28] (Figure 3).

Within the MAP3K family stands out ASK1, which is influenced by this redox state sensor mechanism. ASK1 is crucial in the cellular response to several types of stress. Human and murine proteins are composed of 1374 and 1379 amino acids, respectively; they possess an intermediate domain composed of a serine and a threonine that grant them the kinase activity and are activated by diverse stress factors, among them ROS, lipopolysaccharides (LPS), the tumor necrosis factor (TNF), and calcium input. Nonetheless, it is relevant to mention that the most important stimulus is given by the presence of ROS [15,18]. Under normal redox conditions, human TRX1 with Cys32 and Cys35 in its reduced form interacts directly with the N-terminal region of ASK1; this binding inhibits the kinase activity, hindering the downward signaling cascade. Faced with changes in the redox state by the presence of high ROS concentrations, TRX1 is oxidized and dissociated from ASK1. Once ASK1 is released, the phosphorylation of threonine occurs in its active site (Thr838 in humans and Thr845 in mice), recovering its kinase activity and activating the downward signaling cascade [15]. As with TRX1, the TRX2 also binds to ASK1, inhibiting apoptosis [15,18] (Figure 3). Interestingly, another ASK1 regulation mechanism by TRX1 has been described, which is independent from its function as redox sensor; in this mechanism, TRX1 is proposed as a promoter of ubiquitination and degradation of ASK1, interrupting the ASK1-dependent signaling pathway [15,32].

The relevance of the correct ASK1 regulation in cells is evidenced in the study with ASK1-deficient murine models; in these, an increase in the resistance to apoptosis induced by oxidative stress and a reduction in the sensitivity to the septic shock induced by LPS have been observed. In cultures of ASK1-deficient splenocytes, a diminution in the production of inflammatory cytokines, such as TNF, interleukin 6 (IL-6), and IL-1β, has been observed, suggesting that the ASK1/p38 axis exerts a critical role in the innate immune response. It has also been observed that the activation of the ASK1/p38 pathways induced by LPS diminishes if a pretreatment with antioxidants is provided, indicating that the LPS-dependent ASK1 activation is also mediated by the presence of ROS [15]. Besides the described interactions between ASK1 and TRX1, a protein complex constituted by the endogenous form of ASK1, TRX1, and ASK2 has been identified; this protein complex is called the ASK1 signalosome. Activation of this complex depends on the homo-oligomerization of ASK1 through its C-terminal coiled-coil domains. Its main function is to be a signaling complex that is activated in the presence of ROS. The presence of ROS establishes an oxidant environment, causing oxidation of TRX1, its subsequent dissociation from ASK1, and the recruitment of receptors named tumor necrosis factor receptor-associated factor 2 (TRAF2) and tumor necrosis factor receptor-associated factor 6 (TRAF6) [15].

TRAF2, aside from being an intermediate of the TNF-induced activation of NF-κB, functions as a protein that connects the TNF receptor with its signaling molecules. TRAF2 is associated with a MAP3K that activates the JNK pathways, as occurs also with ASK1 and MEKK1. In turn, TRAF6 is a critical regulator of NF-κB and the MAPK route during the activation mediated by the superfamily of the TNF receptor and of the family of Toll/IL-1 receptors. TRAF6 is also recruited to the ASK1 signalosome by the presence of H_2_O_2_, suggesting that the ROS-dependent interactions of TRAF6/ASK1 are crucial for the activation of ASK1 induced by LPS [15].

Another example of the TRX1 regulation on the signaling cascade is found in the human phosphatase and tensin homolog on chromosome 10 protein (PTEN10), a 54 kDa protein known to be a suppressor of tumors and to function as a phosphatidylinositol-3,4,5-triphosphate capable of regulating cellular processes through its antagonist role to the phosphatidylinositol 3-kinase signaling. The PTEN10 avoids activation of the next step in the signaling cascade by hindering Akt, also known as protein kinase B, from undergoing the needed phosphorylations for its activation (phosphorylation at Thr308 and Ser473). In turn, Akt is a serine/threonine kinase involved in important cellular processes, such as survival, cell proliferation, angiogenesis glucose metabolism, and apoptosis; its activation is a fundamental process for the phosphorylation and activation of proteins, such as caspase 9, the NF-κB factor, and the protein Bcl-2-associated death promoter (BAD), which inhibits apoptosis [15,32] (Figure 3). These interactions between PTEN10 and Akt are modified by changes in the redox state. In the presence of ROS, PTEN10 is inactivated because of the formation of intramolecular disulfide bonds in its active site; in consequence, the PtdIns (3,4,5) P_3_ activates its target in the signaling pathway Akt [15]. In this sense, TRX1 participates as a regulator of the PTEN10 activity because the presence of its reduced form induces the formation of a disulfide bond between Cys32 of TRX1 and Cys212 of the C2 domain of human PTEN10, inhibiting the lipid-phosphatase activity of PTEN10 that avoids, through a steric effect, the binding to the phosphatase site [15].

An additional example of protein kinases regulated by TRX1 is in the protein kinase C (PKC), a kinase of the phospholipid-dependent serine/threonine kinases family that is involved in signaling pathways regulating cell growth, apoptosis, and response to stress. The PKC function is dependent on calcium and is mainly stimulated by a second messenger, diacylglycerol. However, it is also susceptible to the presence of ROS, the possible mechanism involves the selective oxidation of a cysteine-rich region contained in the regulator domain [15]. Some PKC subtypes can interact with TRX1, causing, in the process, an inhibition of the kinase activity of PKCs. The wide range of cell processes that are regulated by the MAPK signaling cascades can provide an idea about the variety of cell functions that can be modified due to the presence of ROS and the direct action of TRX1 [15].

## 10 Regulation of the immune response

In the immune response, TRX1 has also been shown to possess regulating functions with similar behavior to that observed for cytokines and chemokines. *In vitro* experiments suggest that TRX1 possesses co-stimulating activity with TNF, increasing the production of IL-6 and IL-8. It possesses chemoattractant functions for monocytes, neutrophils, granulocytes, and T lymphocytes. TRX1 activates monocytes and macrophages, is a growth factor for lymphoid cells, has a synergistic effect with IL-1 and IL-2, functions as a possible resistance mechanism against HIV-1, because TRX1 secreted by CD4^+^ lymphocytes, and reduces the disulfide bond on the domain 2 of CD4, which is important for the entry of the virus [4,8,31]. In human macrophages activated to M1, the presence of TRX1 diminishes the markers tied to the inflammatory phenotype, such as IL-1β, TNF, IL-6, and IL-8. Likewise, TRX1 increases the differentiation of macrophages to the M2 phenotype; this effect has also been observed in macrophages from rat supplemented intravenously with TRX1 [8,19,33].

An alternative effect to that mentioned previously is in murine peritoneal macrophages; in them, the presence of TRX1 does not induce a change in the activation state of macrophages but exerts an amplifying effect of the activation process to the M2 phenotype. This phenomenon is a consequence of the increment in the production of IL-10 and CD206 interleukin markers (related with the M2 activation state) together with a diminution in the TNF and MCP-1 expression (related with the M1 activation state). The involved mechanism suggests the diminution in p16 expression, favoring the induction of macrophages toward the M2 phenotype, aside from the reduction in the AP-1 and Ref-1 expressions, which makes the macrophages less responsive to the activating stimuli of the M1 phenotype [8].

An important immune regulation mechanism performed by TRX1 is that observed on IL-4; in this case, TRX1 reduces one of the three essential disulfide bonds for the IL-4 function, the one formed between Cys46 and Cys99. Rupture of this disulfide bond leads to the inactivation of IL-4, generating a negative regulating effect on the polarization of macrophages toward the M2 phenotype; this effect is specific for IL-4 and does not affect IL-13 [19].

The study of cultures stimulated with migratory inhibitory factor (MIF) allowed determining that TRX1 binds to this factor, allowing for the formation of a protein complex that fosters the internalization of the extracellular MIF. Possibly mediated by the cluster differentiation 74 (also named major histocompatibility complex class II invariant chain), the chemokine (C–X–C motif) receptor 2 (CXCR2), or the chemokine (C–X–C motif) receptor 4 (CXCR4) of the cell surface. Another level of regulation of the MIF activity could extend to the intracellular level, because for MIF to function as a cytokine it requires the formation of a homotrimeric complex linked through intermolecular disulfide bonds; this suggests that the cytokine activity of MIF could be regulated by TRX1, dissociating the complex [10].

The chemotactic effect of TRX1 has been observed in the respiratory airways of rat, where its administration caused a 41% increase of neutrophils, a value quite above the values usually recorded in individuals without treatment (0.6%). This chemoattractant effect originates in the increased expression of TNF, the chemokine (C–X–C motif) ligand 3 (CXCL3), and the chemokine (C–C motif) ligand 20 (CCL20); being the increase in CXCL3 and CCL20 the key chemotactic factors [31]. At the level of the innate immune response, TRX1 could be functioning as a modifier of the mucosa’s viscosity in the respiratory airways through the reduction in the intermolecular disulfide bonds among the proteins that constitute the mucosa, causing the rupture of the bonds and diminishing the viscosity of the mucosa [31].

**Figure 4:**
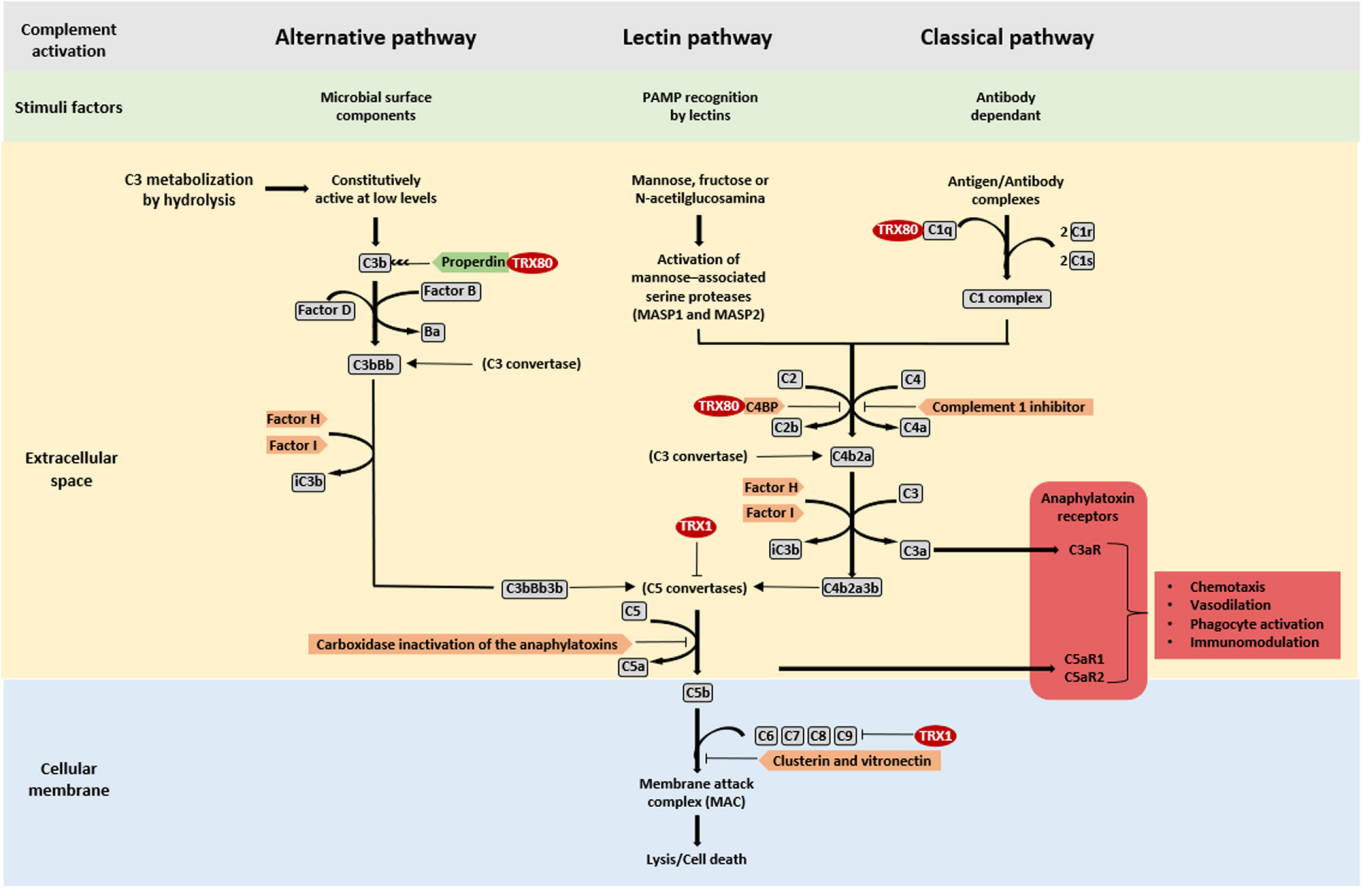
TRX1 and thioredoxin 80 (TRX80) effects on the complement system and proinflammatory polypeptides production. TRX1, by interfering at the level of the C5 convertase and C9, inhibits the activation of the complement system, avoiding the formation of the MAC. In contrast, TRX80 acts as an activator of both the classical and the alternative pathways. In the classical pathway, TRX80 promotes the formation of complex C1 through activation of C1q; likewise, the binding of TRX80 to the C4-binding protein (C4BP) interferes with its inhibiting effect. In the alternative pathway, TRX80 binds to properdin, increasing its activity (sole positive modulator of the complement system). Aside from modulating the complement activation, TRX1 and TRX80 exert important effects on the inflammatory process by modifying the production of two important anaphylatoxins (C5a and C3a), which, through signaling with their respective receptors (C5aR and C3aR), become involved in processes like chemotaxis and activation of cell groups, such as granulocytes, mastocytes, and macrophages.

Another mechanism of the innate immune response mediated by TRX1 is the modulation of the complement at the endothelial level. The complement system is constituted by a series of proteins located in the serum that are activated in sequence to promote the formation of a protein complex called the membrane attack complex (MAC). MAC’s main function is the formation of channels in the cellular membrane that allow for the free diffusion of solutes between the cell cytoplasm and the external environment, which leads to the loss of cell homeostasis and, eventually, death. In this sense, TRX1 inhibits the activity of the C5 convertase (interfering with the production of C5a) and inhibits the activation of C9, hindering the formation of MAC. These inhibitory phenomena are interesting because they are not observed in the presence of TRX80 nor of other proteins of the TRX family, despite presenting the –CXXC– active site [11] (Figure 4).

The above examples point out the potential of TRX1 as a target or as a basis for the implementation of treatment strategies for diverse immunological diseases and alterations. However, it is important to analyze in more detail some of the unresolved mechanisms, like, for example, the cellular uptake mechanism. Regarding this topic, TRX1 uptake is a process of specific and saturable binding, suggesting the presence of some receptor or specific binding site not identified yet. Regarding kinetic details of the uptake process. The extracellular TRX1 is shown to attach rapidly to the surface of macrophages and, at 24 h, it can be located mainly in the lysosomal compartments [8].

## 11 TRX80

TRX80 was identified as a factor that increased the eosinophils toxicity; therefore, it was named eosinophilic cytotoxicity enhancing factor. Human TRX80 is mainly produced by monocytes, it is 20 times more active than TRX1, it has been detected in the plasma at lower concentrations than those recorded for TRX1, and it must be pointed out that the concentrations of both proteins are not correlated. TRX80 is the product of an enzymatic cleavage performed on TRX1 between amino acids 80 and 84, losing its terminal motif αβ and hindering it to function as a substrate for TRXR; in addition, it lacks redox activity [10,34]. TRX1 is the precursor of TRX80 and both are secreted even in the absence of a peptide signal. Up to now, it has not been possible to define accurately the mechanisms involved in the synthesis and the secretion of TRX80. The site where the cleavage originating TRX80 is performed is not known nor the protein in charge of performing that cleavage. A possible secretion mechanism of TRX80 was established in the treatment of monocyte cell lines with an inhibitor of alpha secretases (phorbol ester) enhancing the intracellular concentration of TRX80 and TRX1. Among the alpha secretases are members of the family of A disintegrin and metalloproteinase (ADAM) proteins. To this regard, the combined inhibition of ADAM10 and ADAM17 induces a higher inhibitory effect on the secretion of the different forms of TRXs (≈54% inhibition). Co-localization of signals, observed microscopically, of ADAM17 and TRX1 in the cytoplasm, near the nucleus, and within very focalized zones led to the hypothesis that the route involved in the secretion of TRX1 and TRX80 is not a classical route. The experiments performed in monocytes revealed that the production of TRX80 from TRX1 could involve the ADAM10 and ADAM 17 proteins because the administration of a stimulator like phorbol 12-myristate 13-acelate induces overexpression of TRX80 and of both ADAM proteins [10,33,35].

Besides the role of ADAM proteins in the secretion of TRXs and TRX80, it is important to consider the possible involvement of proteins like those involved in the oxidative folding of proteins as secretion mechanisms [36,37]. Since the secretion of TRXs is dependent on factors, such as the change of the redox state, its localization in the cytosol and the cell surface, the presence of cysteines that form disulfide bridges, and the involvement of enzymatic systems sensitive to redox changes, such as those involved in the oxidative folding of proteins. This suggests the possible existence of similar mechanisms in mammals that facilitate the transport of TRXs across the membrane [38,39].

Among the main functions described for TRX80 are its performance as a cytokine that stimulates the proliferation of monocytes (this effect has not been observed in B or T lymphocytes), as a chemokine for monocytes, T cells, and polymorphonuclear cells, and as a stimulator to produce proinflammatory cytokines [11,27,33,34]. Monocytes are the main cell group influenced by the effect of TRX80, causing in them the expression of surface receptors, i.e., pattern recognition receptors, CD1a, and the mannose receptor, which are essential for the activation of T lymphocytes and the production of inflammatory cytokines. TRX80 also increases the expression of the CD14 monocytes marker and the CD40 activation marker, essential molecules for the activation of T cells, such as CD54 and CD86; it also enhances the pinocytic activity and the production of Th1-type cytokines, such as TNF, IL-1β, and IL-6. In addition, TRX80 presents a synergistic effect with interleukin 12 (IL-12), increasing the production of IL-12 and IFN-γ [27,33,34]. The increase in CD14 expression on the surface of monocytes is relevant in processes of intracellular infections where it allows cells to limit efficiently the proliferation of intracellular bacteria by avoiding their escape from the phagosomes [34].

Although TRX80 lacks the inhibiting effect of TRX1 on the activation of the complement, its activating effect on the complement pathway, both in the classical and alternative routes, must be pointed out. This activating phenomenon is due to the direct interaction with C1q, properdin, and C4BP, causing the deposition of early factors in the complement activation pathway (C4b and C3). A result of this activation of the complement system is the production of anaphylatoxins C5a and C3a, which can bind to their corresponding cellular receptors, triggering effects, such as chemotaxis, vasodilation, activation of phagocytosis, and immunomodulation [11] (Figure 4).

## 12 Role of TRX1 in cancer

The study of the TRX/TRXR system in cancer cells has led to identify the direct relation between its overexpression and the aggressiveness of some types of cancer. In these cases, high TRX1 concentrations in blood during the disease have been observed, to record later normal values after excising the tumor, which emphasizes the relevance of TRX1 in the survival of tumor cells. Another example of the relevance of TRX1 is in the blocking of its expression in tumor cells, which causes the cells to behave less aggressively and even grow in cultures in the same way as would normal cells [9,18]. In the primary gastric cancer exists a significant correlation between the increase in TRX1 expression and the inhibition of the apoptotic process [32]. On the other side, the presence of TRX1 stimulates the angiogenesis induced by the vascular endothelial growth factor and, in some types of cancer, there is a diminution in the amount of the natural inhibitor of TRX1 (TXNIP), making the TRX1 unable to dissociate from ASK1 and hindering the cell to undergo apoptosis by stress [6,9]. The afore mentioned led to establish that TRX1 could be a potential target to tackle certain types of cancer as one of the main mechanisms that foster the survival of tumor cells and their escape from apoptosis.

## 13 TRX1-derived peptides and their therapeutic uses

Involvement of TRXs in many cellular processes and homeostasis turns it into a relevant molecule for the development of treatment strategies of diverse diseases. Among these strategies are the synthesis of mimetic peptides, tri- or tetra-peptide sequences like the canonic sequence of the active redox site of TRXs blocked in both ends by an *N*-acetyl group and an amide group. This design provides pertinent therapeutic advantages, such as easiness to permeate the inside of cells, the possibility to cross through the blood–brain barrier, and the ability to function as reducers of proteins that are usually the target of TRXs and to function as direct antioxidants in the neutralization of ROS [29,40,41]. Examples of these peptides are the CB3 (NAc-Cys-Pro-Cys-NH_2_), CB4 (NAc-Cys-Gly-Pro-Cys-NH_2_), and CB6 (NAc-Cys-Gly-Ala-Cys-NH_2_) peptides, which function as factors that reverse the oxidative stress in models of stress induced by the inhibition of TRXR in rats, being CB3 and CB6 the peptides with the highest activity [29,40,41]. These peptides, like TRX1, can inhibit the activation of ASK1, avoiding the activation of the MKK4/7-JNK and MKK3-p38 signaling pathways. Besides, they diminish the phosphorylation of ERK1/2, associated with the stress response-signaling pathway [40].

On the other side, peptides CB3 and CB4 have shown denitrosylation activity for several types of substrates by accepting a NO group from the substrate, forming the corresponding nitrosylated intermediaries (CB3-SNO and CB4-SNO). Aside from this denitrosylated activity, peptides also function as accelerators of the enzymatic reduction of nitrosylated proteins because both, the nitrosylated and the oxidized forms of peptides, are regenerated to a reduced state by TRXR and proteins with NADPH-dependent oxide-reducing thiols, in this way, joining the global process of enzymatic reduction [29]. Because of their denitrosylating activity, CB3 and CB4 protect the function of PRX and TRXR by avoiding their nitrosylation. This becomes relevant in diseases like Parkinson’s disease, where the high level of nitrosylation of PRX2 increases the vulnerability of neuronal cells to oxidative stress, or Alzheimer’s disease where the high nitrosylation levels of PRX1, PRX3, and PRX6 are factors that increase the oxidative stress in neuronal cells [29].

In addition, CB2 peptide administration in murine asthma models prevents the damage caused by ROS by inhibiting the activation of p38 and, thereby, avoiding the activation and nuclear translocation of the NF-κB factor [29,41]. Both CB3 and CB4 have been demonstrated *in vivo* and *in vitro* to diminish the oxidative stress in cells by inhibiting the phosphorylation of JNK and p38, avoiding the translocation of NF-κB to the nucleus. In diabetic rat models (Zucker diabetic fatty rats), CB3 diminishes the phosphorylation and activation of the MAPK signaling route, which diminishes the apoptotic process. In a model of human neuronal cells (SH-5Y5Y), CB3 and CB4 diminish excision of caspase 3, preventing the dissociation of PARP and reversing the phosphorylation of JNK induced by TNF [41]. In the diabetic rat model, administration of CB3 diminishes the levels of TXNIP through a mechanism not-determined yet, but which is possibly mediated by the presence of ROS. This diminution causes an increase in the sensitivity to insulin in cells and the secretion of insulin induced by glucose during diabetes, suggesting a possible therapeutic alternative for type 2 diabetes [41].

## 14 Conclusions

**Figure 5:**
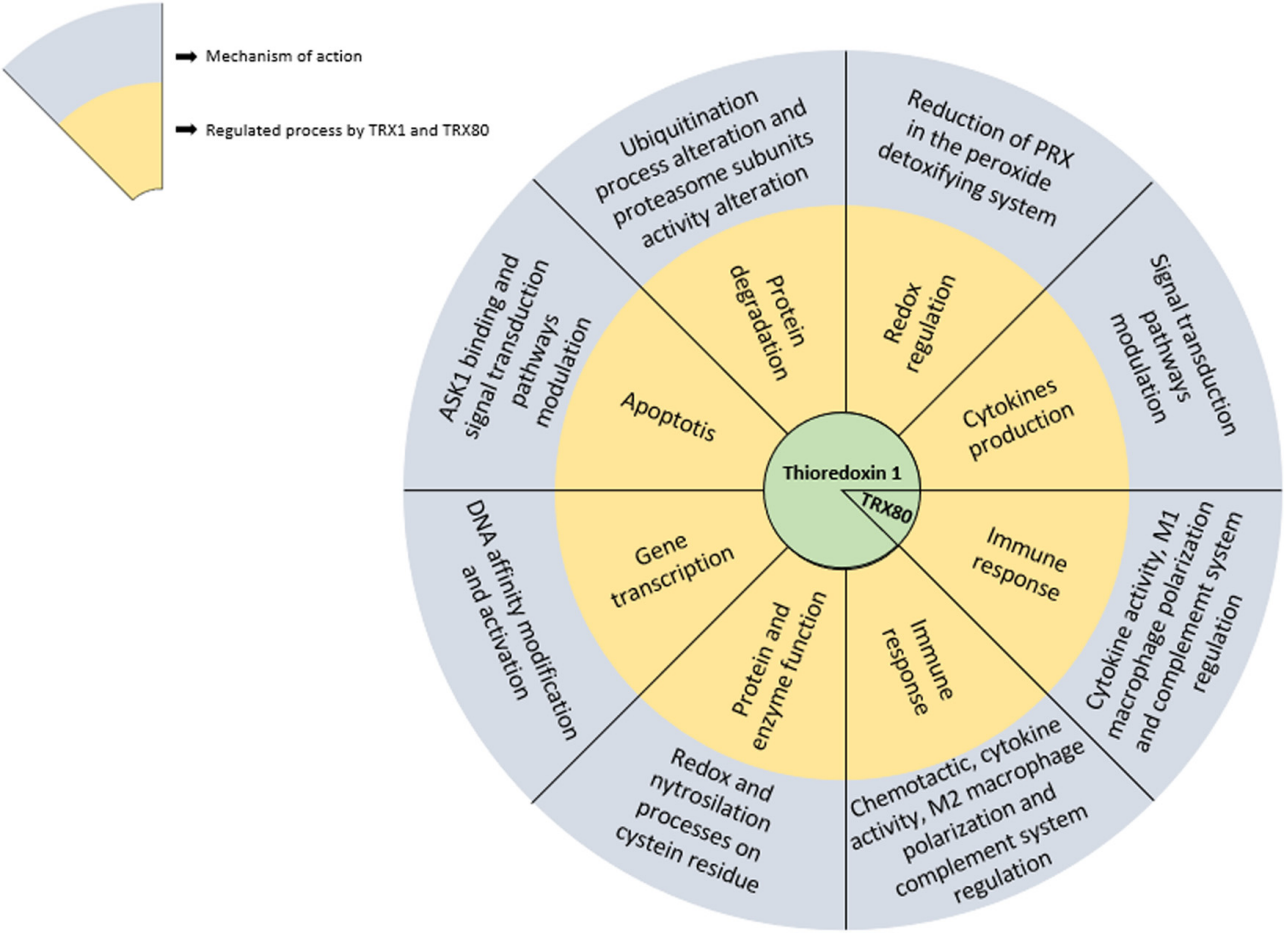
Participation of TRXs in different cell processes, diseases, and possible applications. Due to their redox properties, transnitrosylation ability, chemotactic effects, or their cytokine functions, TRXs participate in a wide and varied number of cell processes. This figure emphasizes the physiological relevance of TRX1 in some types of cancer where they act as a survival factor, in inflammatory processes, such as atherosclerosis, Alzheimer’s disease, Parkinson’s disease, type 2 diabetes, and obesity. These data raise the development of possible therapeutic strategies, either using TRXs as target for the treatment of diseases or as therapeutic factors; in the latter case, TRX-derived peptides have been developed, which have shown a redox regulatory activity as well as an immunomodulatory capacity.

TRXs, through their redox properties and S-nitrosylating function, regulate the activity of many cellular processes needed for cell homeostasis, the general redox state of cells, and the modification of proteins and their functions. The inadequate performance of the TRX system compromises importantly cell homeostasis through either the loss of regulating mechanisms or establishing an uncontrolled oxidative stress state (Figure 5). The foregoing highlights the physiological relevance of TRXs and allows proposing strategies for the treatment of different diseases in which TRX1 is the main axis. For example, fostering its overexpression can diminish the damage associated with inflammatory processes. In cancer, TRX1 is one of the factors that allow cells to escape apoptosis; hence, diminishing its activity could contribute to decrease the proliferation of this type of cells.

The type of modifications performed by TRX1 and the large number of target proteins that it contains place TRXs as a very relevant group of proteins for the study of many cell mechanisms and point to it as a possible candidate for the development of treatment strategies for diverse diseases. The continuous understanding of their functions and interaction mechanisms have allowed developing smaller molecules capable of performing their functions and representing important therapeutic advantages, like their greater permeability to the cell. Notwithstanding, there are several mechanisms that must still be detailed. For example, the unknown mechanisms involved in the secretion of TRX1 in the surface of cells (like phagocytic cells), the identity of the protein in charge of performing the cleavage of TRX1 that gives origin to TRX80, or the cellular site where this process is accomplished. Knowledge of these mechanisms would allow developing better strategies to modify specifically the production of these proteins aimed at influencing the described cell processes.
